# 1525. Evolving Trends in HIV Transmitted Drug Resistance in Eastern North Carolina

**DOI:** 10.1093/ofid/ofad500.1360

**Published:** 2023-11-27

**Authors:** Srinivas Reddy, Jacob Pierce, Hui Bian, Dora Lebron

**Affiliations:** East Carolina University, Greenville, North Carolina; Brody School of Medicine at East Carolina University, Greenville, North Carolina; East Carolina University, Greenville, North Carolina; East Carolina University, Greenville, North Carolina

## Abstract

**Background:**

The impact of HIV/AIDS worldwide is an important public health challenge. One factor that contributes to increased HIV transmission is HIV drug resistance. This occurs from the intrinsic nature of HIV to mutate and eventually replicate in the presence of antiretroviral therapy. The effects of this are development of treatment failure and further transmission amongst cohorts in a given population. The problem of propagated drug resistance is further compounded in populations that present with problems of adherence. A previous study conducted at our institution demonstrated no association with race and transmitted drug resistance.

**Methods:**

This is a retrospective study that evaluated treatment-naive HIV patients with genotype results from 2016-2020 at East Carolina University infectious disease clinic in Greenville, NC. We collected data on age, race, sex, HIV risk factor, and genotype. A logistic regression model was fit for transmitted HIV resistance with the following covariates: age, gender, race, year, and men who have sex with men (MSM) status. Individual comparisons were made by chi-squared testing for categorical variables or Wilcox ranked sum test for non-normally distributed continuous variables. All observations are presumed to be independent. All data analysis was performed in SAS (SAS Institute Inc., SAS 9.4, Cary, NC: SAS Institute Inc., 2002-2023).

**Results:**

Of 305 charts reviewed, 245 were included in the analysis. Twenty-six (13.7% African-Americans) versus 14 (25.9% of other races) were found with transmitted drug resistance. Twenty four (16.4%) were MSM. The years with higher transmitted drug resistance detected were 2017-2019 (18.7%, 16.5%, 17.1%, respectively). African American race was found to be protective against transmitted resistance (OR 0.46, 95% CI 0.22-0.98, p = 0.04) when adjusting for MSM status, Sex, Age, and Year. The result was also significant in the unadjusted analysis (χ^2^ = 4.60, p = 0.03). Non-African Americans were 2.17 times more likely to have transmitted resistance than African Americans.

**Table 1:**
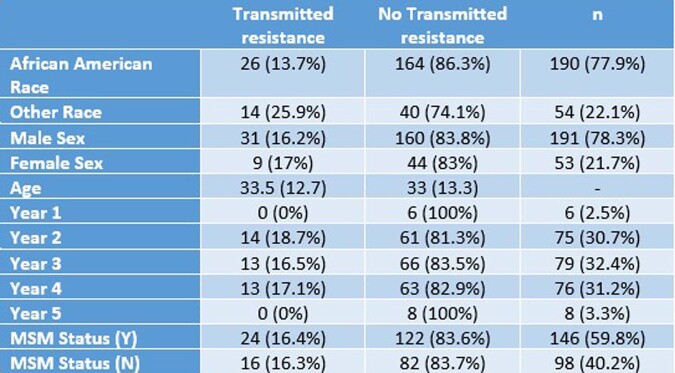
Patient Characteristics. Patient characteristics presented as proportion and percentage for categorical variables and median and standard deviation for continuous variables.

**Table 2:**
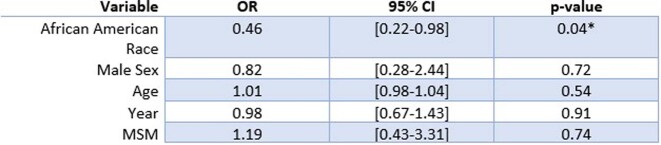
Adjusted logistic regression for variable effect on transmitted antiviral resistance

**Conclusion:**

African American race was found to be protective against transmitted resistance (OR 0.46, 95% CI 0.22-0.98, p = 0.04) when adjusting for MSM status, sex, age, and year. This is a change from the results of our previous study.

**Disclosures:**

**All Authors**: No reported disclosures

